# Moderated Online Social Therapy (MOST) in Help-Seeking Young People: Pilot Randomized Controlled Study

**DOI:** 10.2196/73269

**Published:** 2025-11-21

**Authors:** Maeve Dwan-O'Reilly, Sophie Mae Harrington, Conor Gavin, Emmet Godfrey, Megan Cowman, Christina Gleeson, Anna O’Mahony-Sinnott, James McCormack, Emma Frawley, Tom Burke, Karen O'Connor, Max Birchwood, Caroline Heary, Mario Alvarez-Jimenez, Gary Donohoe

**Affiliations:** 1 School of Psychology, Ollscoil na Gaillimhe – University of Galway, University Road, Galway, Ireland, 353 91 524411; 2 Centre for Neuroimaging, Cognition, and Genomics (NICOG), Ollscoil na Gaillimhe – University of Galway, Galway, Ireland; 3 Student Counselling Service, Ollscoil na Gaillimhe – University of Galway, Galway, Ireland; 4 RISE HSE Early Intervention in Psychosis Service, Cork, Ireland; 5 Department of Psychiatry and Neurobehavioural Science, University College Cork, Cork, Ireland; 6 Warwick Medical School, University of Warwick, Coventry, United Kingdom; 7 Orygen, Parkville, Victoria, Australia; 8 Centre for Youth Mental Health, The University of Melbourne, Melbourne, Australia

**Keywords:** mental health, young adult, digital mental health intervention, social therapy, university, pilot

## Abstract

**Background:**

In the context of a sharp rise in help-seeking in youth mental health, digital mental health interventions offer enormous potential to improve outcomes, facilitate access, and meet the increasing demand for mental health services. For example, for young adults attending third-level education, digital mental health interventions may support help-seeking students while either waiting to attend student counseling or to sustain gains once a brief course of face-to-face counseling sessions has been completed. Moderated Online Social Therapy (MOST) is an online intervention that combines tailored psychotherapeutic content with one-to-one therapist and peer support worker support, and an online community. MOST has an emerging evidence base in multiple mental health contexts, but it has not yet been implemented in the university context.

**Objective:**

This trial investigated the feasibility of using MOST to support the mental health of third-level students who recently attended a student counseling service.

**Methods:**

We conducted a pilot randomized controlled study of third-level students who had recently completed ~4 sessions of counseling in their university counseling service. Students were randomly assigned to the intervention or control arm at a rate of 2:1. In the intervention arm, students had access to MOST for 26 weeks, and both groups were assessed at baseline, 12 weeks, and 26 weeks. Outcomes assessed at each time point included social and occupational functioning, cognitive functioning, depression, anxiety, and loneliness. To examine the feasibility of the trial, we examined data on recruitment, trial retention, and engagement with the MOST platform. We calculated effect sizes for outcome variables to explore the preliminary efficacy of the MOST intervention.

**Results:**

A total of 74 participants were recruited, meeting the recruitment target of ~3.1 participants per semester month. Retention in the trial was 70% (52/74) at 12 weeks, reducing to 66% (49/74) at 26 weeks. For the intervention group, when engagement was measured in terms of participation in at least one component of the intervention, 81% (38/47) of the intervention group engaged for 5 or more weeks of the trial (~20% of the maximum 26 weeks). Based on the effect sizes observed, the intervention arm was associated with modest gains in social function and cognitive function and reduced clinical symptom severity at 12 weeks.

**Conclusions:**

Based on the recruitment, retention, and engagement rates observed, a full randomized controlled trial of MOST with young adults at the university is feasible. Moreover, the effect sizes favoring the intervention arm are consistent with previous studies and support a full trial of MOST as a potentially beneficial support for youth mental health in further education settings.

## Introduction

### Digital Interventions in Youth Mental Health

Despite having the greatest level of need, young adults have the worst access to timely and quality mental health care [1]. Both before and after the COVID-19 pandemic, there is robust evidence that demand for youth mental health support significantly outstrips availability in both the health care and education systems [2]. In the context of a sharp rise in help-seeking, digital health interventions (mental health supports that are delivered via web-based or mobile-based platforms) offer enormous potential to improve outcomes, to widen access, and to meet the increasing demand on mental health services.

Several meta-analyses have been conducted on digital interventions (mostly focused on cognitive behavioral therapy [CBT] or “third-wave” cognitive interventions) that address depression and anxiety in young adults. An umbrella review by Harith and colleagues [3] found evidence to support the use of digital interventions, but noted that effectiveness was greatly dependent on the delivery format and the mental health problem targeted. Furthermore, Harith et al [3] noted that despite young people (as “digital natives”) frequently expressing a preference for the internet as a source of seeking health-related information to address or solve health problems, engagement with and adherence to digital health interventions is often suboptimal.

### Moderated Online Social Therapy

One approach to improving mental health recovery in young adults is Moderated Online Social Therapy (MOST) [4]. MOST was initially developed as a digital mental health platform to provide a low-intensity, cost-effective, and engaging approach to prolonging the benefits of specialized Early Intervention for Psychosis (EIP) services [4]. MOST has shown benefits in terms of return to education and employment among participants, decreased need for emergency care, and has shown to be cost-effective from both the health care sector and societal perspective [5]. MOST has since been trialed in young adults in single-arm studies of help-seeking young people aged between 16 and 25 years in Australia [6] and the Netherlands [7], and in young people with depression [8], social anxiety [9], at high risk of psychosis [10], at increased risk of suicide [11], with borderline personality disorders [12], and in a large-scale national study of young adults in Australia [13], with small to large benefits observed for social function and symptom severity. As a digital intervention, MOST consists of both evidence-based online therapy content supported by therapist contact and a Facebook-style community supported and moderated by peer support workers. Evidence of the acceptability of MOST for young people has been reported in a number of studies, including in young people with social anxiety [14], emerging mental health issues [7], and psychosis [15].

The design and therapeutic content of MOST is strongly influenced by self-determination theory [16], an empirically supported theory of motivation, which focuses on the processes and social environments that facilitate or hamper social functioning. In terms of engagement and adherence, MOST differs from other “self-help” style digital interventions by providing access to therapeutic content online that is supported by access to a therapist. It is further supplemented by social supports, including peer support and an online community. Providing these face-to-face supports is likely to improve engagement, which is identified as a major barrier to the use of digital interventions [7,17,18].

### Student Mental Health

In many European countries, young adults remain in some form of education until their early twenties. Based on the figures published in 2022 by the Organisation for Economic Co-operation and Development, 54% of 18‐ to 24-year-old young adults are in some form of third-level education, rising to 59% in Europe, and up to 63% in Ireland [19]. According to a 2018 World Health Organization study of ~14,000 students from across 8 countries, approximately one in three screened positive for at least one common DSM-IV (*Diagnostic and Statistical Manual of Mental Disorders*, Fourth Edition) anxiety, mood, or substance disorder [20]. Similarly, Sheldon et al [21], in a meta-analysis of third-level students, reported a pooled prevalence of depression at 25% and suicide-related outcomes at 14%. Taken together, these data suggest that education settings such as universities and colleges represent a key location for the development and delivery of mental health interventions. As noted above, however, access to young adult mental health services is often limited, and particularly so in university mental health services, where a student may at best have access to short-term (1-4) sessions of counseling. In this context, MOST may provide a means to supplement existing 1:1 therapy in a scalable and cost-effective manner.

### Objectives

The purpose of this study was to provide information about the feasibility of conducting a randomized trial of MOST in young people who recently attended a university counseling service, along with preliminary data regarding the efficacy of MOST for the purpose of a definitive randomized controlled trial. This study was carried out as part of a funded program of research entitled “Improving Psychosocial Supports in Youth Mental Health” (the PSYcHE program).

## Methods

### Ethical Considerations

This study was approved by the Galway Clinical Research Ethics Committee, Merlin Park Hospital, Galway, Ireland (reference CA2468). All participants provided informed written consent, and protocols were put in place for the proposed management of vulnerable individuals in the study. Participants were reimbursed €25 (approximately US $29) for each assessment (see below). The ethics application also detailed General Data Protection Regulation (GDPR) considerations including the pseudonymization of data and data management practices to ensure the privacy of participants. The trial was registered with ISRCTN (number 15520701).

### Setting and Inclusion Criteria

We conducted this prospective, assessor-blind, randomized controlled pilot study at the student counseling service of the University of Galway, Ireland, which serves just over 18,000 students. We aimed to include students who attended the service with persistent mental health difficulties of at least 12 months in duration. The rationale for this was to focus on a more homogeneous sample of young people seeking help for mental health difficulties rather than more transient difficulties causing distress. Inclusion criteria were being aged between 18 and 35 years, self-reporting mental health difficulties of longer than 1 year in duration, being clinically stable, and having the ability to give consent. Clinical stability in this context was determined by the referring counselor. A participant was considered eligible for MOST based on the criteria that the participant was finishing their attendance at the counseling service and no longer required the 1:1 counseling support they had been receiving. Exclusion criteria were a history of organic impairment (including IQ<70), a history of head injury with loss of consciousness >5-minute duration, and substance abuse in the preceding month.

Referrals to the study were made electronically by the student counselor toward the end of the participant’s short-term 1:1 counseling sessions (~4 sessions were provided). Once the referral was received, a research assistant completed the informed consent procedure, after which an initial screening was conducted. Following screening against inclusion and exclusion criteria, eligible participants were enrolled into the study and completed a baseline assessment. See Multimedia Appendix 1 for a breakdown of recruitment across the study period.

### Randomization and Masking

#### Procedure

Following baseline assessment, participants were randomly allocated to their treatment group. Randomization was implemented using Sealed Envelope [22] at a rate of 2:1 intervention versus control. A block design approach was taken to account for gender, with 6 participants per block. Research assessors (master’s level psychology research assistants) were masked to group allocation. Participants were informed about their trial allocation by a research assistant who was independent from the research assessors and whose role was to “onboard” participants onto the MOST intervention platform. To ensure that assessors remained blind to treatment status, research assessors were asked to guess the treatment arm of the participant following each assessment. Assessors correctly guessed intervention status only 39% of the time (12/31 guesses made; *χ*^2^=2.62, *P*=.11), suggesting that assessors were in fact blinded to status.

#### Intervention Group

##### Overview

For those randomized to the MOST arm, an onboarding process was completed, during which participants were registered with the platform and given a guided tour. MOST has been described elsewhere [13,23,24]. In brief, participation in the platform consisted of (1) engaging with a therapy “journey”; psychotherapeutic content automatically tailored for each participant based on their response to a questionnaire completed as part of the onboarding, further discussion below, (2) support with participation on the therapy journey from a therapist in the form of fortnightly ~15-minute video or telephone calls, (3) a community wall, see below, and (4) peer support for engagement in the community wall in the form of fortnightly ~30-minute video or telephone calls. Clinical and peer support workers followed established protocols provided by the MOST platform developers. Clinicians and peer support workers also met with the study principal investigator, as a group, on a monthly basis. During these meetings, engagement with each participant was reviewed and assessed according to the therapist and peer support manuals.

##### Therapy Journey

The Therapy Journey took the form of interactive online therapy modules (focusing on anxiety, social anxiety, depression, and social interaction) based on third-wave CBT and primarily targeting social functioning by, for example, fostering self-efficacy (identifying personal strengths based on the strengths-based framework), positive emotions and subjective well-being (eg, practicing mindfulness and self-compassion), or positive connections with others (eg, focusing on empathy skills). Participants’ engagement and application of this content in daily life is supported by 5 activity types: *comics*, *reflective actions*, *actions*, *talking points*, and *pages. Comics* are illustrated multipaneled narratives that bring therapeutic concepts to life via recurring characters, *reflective actions* are prompts for reflection, *actions* suggest a practical step (eg, behavioral experiment), *talking points* prompt young people and peer workers to post their thoughts and reactions to the content, and *pages* summarize each track and provide psychoeducation. Users have the option to save activities to a “toolkit,” so they have an accessible, personalized, and labeled bank of strategies when needed.

##### Community Interaction

The MOST community took the form of an online social network to foster social support. Participants are encouraged to communicate with one another and with peer and expert moderators. This is in order to foster a sense of connection as well as combat loneliness and self-stigma [13]. The community includes a “feed” page that allows participants to post text, images, and links to be read and responded to by other members of the community. This feed is only available to others on the MOST platform and is moderated by clinicians and led by peer-support workers with lived experience and informed by the evidence-based problem-solving framework [6]. A further feature of MOST is an online group function, referred to as “Talk it Out,” which enables users to nominate issues (eg, “how to break through shyness and make new friends?”), which are discussed in moderated groups through structured problem-solving phases (eg, brainstorming, pros and cons, and wrap-up).

### Control Group

Those randomized to the control arm of the study continued to receive care as usual. As participants entered the trial after attending the student counseling service, they were free to seek help from usual supports both internal and external to the university (student medical services, etc). However, control participants were not provided additional supports through the trial, and in a majority of cases, were not receiving other therapeutic intervention during the period assessed. Control participants could be onboarded to MOST following their 26-week assessment.

### Outcomes Assessed

As a pilot study, our main outcome metrics related to the feasibility of the trial included the number of participants recruited, their engagement with the treatment, and their retention at the follow-up assessment period. In addition, we also aimed to establish the feasibility of our primary and secondary outcome measures, along with some indication of treatment effect estimates that might be expected for these measures, to inform power calculations for a full trial.

Outcome measures were administered to participants in both groups by assessors blinded to intervention allocation at baseline, 12 weeks, and 26 weeks. Participants were reimbursed €25 (approximately US $29) for their time for each assessment. The overarching aim of the PSYcHE research program under which this study was carried out was to improve psychosocial function. As such, our main outcome variable was social and occupational function. As described in detail previously [25], identifying suitable measures of social and occupational function is complex, agreeing with the adage that simple measures are not accurate and accurate measures are not simple. As a result, for the purposes of this study, we included two separate measures of social and occupational function. The first was the Social and Occupational Functioning Assessment Scale (SOFAS) [26], an interviewer-rated global assessment of social and occupational function. The second measure was the Time Use Survey (TUS) [27], an interviewer-rated assessment of constructive economic activity and structured activity.

Given that improved social and functional outcomes may relate changes in cognitive and social cognitive function (a hypothesis of the broader PSYcHE program), two measures of cognitive and social cognitive function were included—the Weschler Logical Memory task [28], which is a brief measure of verbal episodic memory, and the Reading the Mind in the Eyes Test (RMET) [29], which is a brief measure of social cognition, measuring theory of mind.

In terms of clinical variables, we initially intended to assess the feasibility of loneliness, as measured by the UCLA (University of California, Los Angeles) Loneliness Scale [30]. However, studies published soon after the start of the trial, both of MOST and of other digital interventions, indicated that some of the largest effects might be observed on measures of anxiety, mood, and distress (eg, [4,11,14]). Consequently, the trial protocol was amended after 6 months to also include additional clinical measures. That is, a measure of anxiety (Generalized Anxiety Disorder-7; GAD-7) [31] and of depression (Patient Health Questionnaire-9; PHQ-9) [32]. These were therefore available for 51 of the 74 participants, 32 in the intervention arm and 19 in the control arm.

### Intervention Engagement Data

Engagement data were extracted from the MOST platform once participants had completed 26 weeks on the platform. Data extracted included time spent, in minutes, working through therapeutic content as well as the number of therapeutic content activities completed, community activity (posts, comments, and reactions on the community wall), and texts and calls with therapists and peer support workers. The number of weeks spent engaging with the intervention was also calculated for each participant. Engagement was estimated as the combined total number of weeks that each participant logged into the MOST platform and moved beyond the homepage, or engaged in a call with their assigned therapist or peer support worker.

Evidence around engagement with digital mental health interventions, including definitions of engagement and thresholds for engagement, is inconsistent and varied depending on the type of digital intervention, the participants involved, and the context in which the intervention is implemented [33-35]. As previous studies have measured engagement in different ways, leading to uncertainty about what engagement could be expected, we did not set an a priori indicator of engagement. In the absence of an agreed-upon measurement of engagement, we adopted a pragmatic approach informed by adherence literature as well as rates of engagement in previous digital interventions [34-36]. As such, we defined engagement as active use of one or more aspects of the MOST intervention and applied the following thresholds: We considered minimal engagement as >20%, partial engagement as 50%, and full engagement as >80%. For a 26-week trial, 20% engagement approximates to engagement for 5 or more weeks, and 50% as engagement for more than 12 weeks.

### Statistical Analysis

Formal sample size calculations were not performed for this pilot study. Instead, the target sample size was based on recruitment of an adequate sample size for a pilot study conducted for the purposes of establishing parameters for a definitive study. Previous guidance on sample size estimation for pilot studies [37,38] has suggested a minimum of 60 participants as an adequate sample. A period of 36 months was initially proposed as a timeframe to recruit participants into the trial. This was based on funding, and also allowing for the fact that the majority of students were on campus for ~7 months of the year. As such, we sought to recruit a minimum average of 3 students per university term month, approximately 20 students per year of the trial.

To assess differences between groups, analyses of covariance (ANCOVAs) were carried out to obtain adjusted mean differences between groups with a 95% CI, while accounting for baseline variables. Effect sizes were also calculated by taking the β coefficients of the treatment arm from the ANCOVAs and dividing them by the pooled baseline SD for each measure, respectively. The analysis plan did not include reporting of *P* values; this was based on recommendations that, as pilot studies are not fully powered, interpretation of results should be done with caution and the analysis should be designed to inform future trials rather than hypothesis testing [39]. Effect sizes were reported using the standard cutoffs of small (*d*=0.2), medium (*d*=0.5), and large (*d*=0.8) [40]. All analyses were completed at the end of the last follow-up assessments and were based on the intention-to-treat population. Analyses were carried out between the intervention and control groups as well as between participants who engaged for 5 or more weeks (minimum threshold for engagement) and the control participants. Analyses were conducted using SPSS v29 (IBM Inc).

## Results

### Recruitment and Sample Description

#### Recruitment

**Figure 1.**
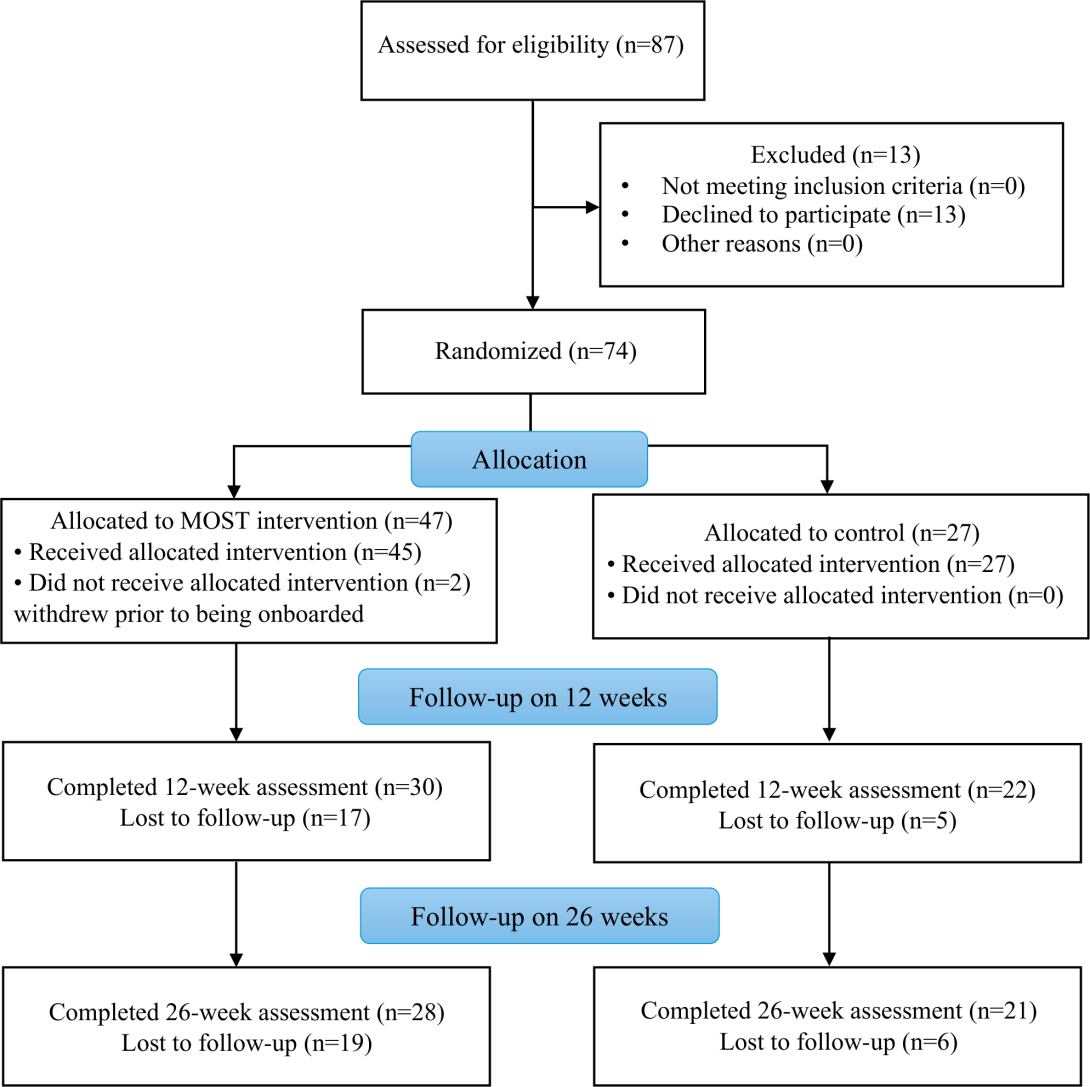
Participant recruitment and retention. MOST: Moderated Online Social Therapy.

Our initial target was to recruit 60 participants over a 36-month period, representing a recruitment rate of 20 participants per year. We expected that most of the recruitment would occur during the 7 months of term time, such that we would be required to recruit ~3.3 participants per month of term (semester) time (~1.67 participants per calendar month) to achieve this target. The actual number of participants recruited was 74 over the 44 months (extended by 8 months due to the COVID pandemic) between April 2021 and December 2024 (see Figure 1 for the CONSORT [Consolidated Standards of Reporting Trials] participant flowchart [41]). In terms of the university semester, this equates to ~3.1 participants recruited for each month of term time or ~1.68 participants per calendar month (see Multimedia Appendix 1 for recruitment numbers by month).

#### Sample Description

A demographic and clinical description of the sample is provided in Table 1. The sample had a mean age of 22.69 years (SD=5.34) and 72% (53/74) identified as female. Nearly 68% (50/74) of the sample lived in shared accommodation, while 24% (18/74) continued to live at home. Predictably, for a sample recruited from a student cohort, all but 2 participants were in full- or part-time education at the time of baseline assessment, with those 2 participants having just left education since initial contact.

**Table 1.** Demographic and clinical description of the sample at baseline assessment.

	Intervention group (n=47)	Control group (n=27)
Age (years), mean (SD)	22.77 (6.12)	22.56 (3.71)
Gender (male:female:genderflux:other)	12:32:1:2	5:21:0:1
Current education status, n (%)		
Not in education	1 (2)	1 (4)
Part time	2 (4)	3 (11)
Full time	44 (94)	23 (85)
Accommodation, n (%)		
Lives with parents	13 (28)	5 (19)
Lives with others	30 (64)	20 (74)
Lives alone	1 (2)	1 (4)
Presenting problem (self-report), n (%)		
Anxiety	20 (43)	13 (48)
Mood	10 (21)	7 (26)
Academic	6 (13)	5 (19)
Relational	4 (9)	1 (4)
Behavioral	3 (6)	0 (0)
Other	4 (9)	1 (4)
Duration of difficulties (months), mean (SD)	44.45 (40.66)	52.33 (44.83)
Clinical measures, mean (SD)		
GAD-7^a^	11.12 (4.77)	11.63 (4.93)
UCLA^b^ Loneliness Scale	47.96 (11.3)	48.04 (10.33)
PHQ-9^c^	12.32 (5.06)	13.63 (5.11)
Risk of alcohol and drug dependency, mean (SD)		
AUDIT^d^	7.29 (6.38)^e^	7 (4.53)
DUDIT^f^	2.26 (4.62)^g^	2.67 (4.53)
Predicted general cognitive ability, mean (SD)		
TOPF^h^	104.16 (8.72)	104.93 (6.63)

a GAD-7: Generalized Anxiety Disorder-7.

b UCLA: University of California, Los Angeles.

c PHQ-9: Patient Health Questionnaire-9.

d AUDIT: Alcohol Use Disorders Identification Test.

e n=45.

f DUDIT: Drug Use Disorders Identification Test.

g n=46.

h TOPF: Test of Premorbid Functioning.

In terms of duration of mental health difficulties, reflecting the inclusion criteria of experiencing difficulties for at least 12 months, participants subjectively reported duration of difficulties as ranging from 12 to 165 months (13.8 years) in the intervention arm and 12‐192 months (16 years) in the control arm. The most commonly self-reported difficulties were anxiety (33/74, 45%), mood (17/74, 23%), academic difficulties (11/74, 15%), and relationship difficulties (5/74, 7%). In terms of alcohol use—measured by the Alcohol Use Disorders Identification Test (AUDIT) [42]—59% (44/74) were classified as “low risk,” 30% (22/74) as “low risk” or “increasing risk,” and 7% (5/74) as “possibly dependent” on alcohol. In terms of drug use—measured by the Drug Use Disorders Identification Test (DUDIT) [43]—all participants were estimated as “probably not” substance dependent and none as probably heavily dependent.

In terms of randomization, which was set at 2:1 intervention to control, 47 (64%) of the 74 participants were assigned to the intervention arm of the study. No randomization errors were identified during the trial period.

### Rates of Retention and Engagement

#### Retention

This was defined as the percentage of participants who completed outcome measures at 12 and 26 weeks. Our initial target for retention was 75% at 12 and 26 weeks, with a threshold target of 70% required to proceed to a full randomized controlled trial without amendment of the study. In terms of actual retention rates in the study, 12-week assessments were collected for 52 (70%) of the 74 participants, representing 30 (64%) of the 47 intervention participants and 22 (81%) of the 27 participants in the control arm. Twenty-six-week assessments were available for 49 (66%) out of the 74 participants. This represented 28 (60%) of the intervention group and 21 (78%) of the control group. In summary, this reflects an overall dropout rate of 30% at 12-week follow-up, increasing marginally to 34% at 26 weeks.

#### Rates of Engagement

As described above, engagement was captured in terms of discrete activities on MOST (see Multimedia Appendix 2) and then categorized in terms of number of weeks of engagement in the intervention. Participants were categorized according to their level of engagement using the following thresholds: minimal engagement ≥5 weeks, 20% of the intervention period; partial engagement ≥12 weeks, 50% of the intervention period; and full engagement ≥21 weeks, 80% of the intervention period. Of the 47 participants in the intervention arm, we observed that 38 (81%) engaged with the program for 5 or more weeks, 39 (83%) were engaged in the first 12 weeks, and 35 (74%) continued to engage beyond 12 weeks (see Multimedia Appendix 3). Finally, to allow future comparison with other studies of MOST and other digital interventions, engagement was also recorded and summarized in terms of min-max; median and mean for total activity time; number of therapy journey activities completed; community posts made, commented on, or reacted to; and number of calls with the therapist and peer support worker. These are provided in Multimedia Appendix 2.

### Outcome Measures

Table 2 shows the group means and SDs, adjusted mean differences, and the effect sizes for those differences between the intervention group and the control group for all outcome measures. Table 3 shows the same descriptive values for participants from the intervention group who had a minimum of 20% engagement on MOST, compared to the control group.

**Table 2.** Comparison of outcome data for full sample.

	Intervention group		Control group		Adjusted mean difference (95% CI)	Effect size (*d*)
	Participants, n	Mean (SD)	Participants, n	Mean (SD)		
Primary outcome measures						
Social and occupational function						
SOFAS^a^						
0 weeks	47	79.38(8.26)	27	77.89 (8.86)	—^b^	—
12 weeks	29	81.48 (9.83)	22	78.45 (10.82)	–2.36 (–8 to 3.27)	–0.28
26 weeks	24	82.79 (10.26)	21	83.52 (7.7)	1.08 (–4.09 to 6.25)	0.13
TUS^c^ constructive economic activity						
0 weeks	47	43.77 (19.13)	27	45.3 (29.52)	—	—
12 weeks	30	36.42 (15.26)	22	48.32 (32.04)	10.86 (–1.66 to 23.37)	0.47
26 weeks	28	38.18 (19.52)	21	42.85 (14.91)	4.36 (–6.06 to 14.78)	0.19
TUS structured activity						
0 weeks	47	53.62 (19.33)	27	53.67 (28.23)	—	—
12 weeks	30	46.23 (16.08)	22	59.63 (31.38)	12.74 (0.66 to 24.83)	0.56
26 weeks	28	47.91 (21.43)	21	52.49 (16.09)	4.13 (–7.08 to 15.34)	0.18
Secondary outcome measures						
Cognitive and social cognitive function						
RMET^d^						
0 weeks	47	27.19 (4.3)	27	27.11 (3.48)	—	—
12 weeks	30	27.1 (4.36)	22	25 (4.16)	–0.93 (–2.67 to 0.81)	–0.23
26 weeks	28	28.21 (4.3)	21	27.48 (3.49)	–0.11 (–1.98 to 1.76)	–0.03
Logical memory^e^						
0 weeks	47	9.38 (2.89)	27	8.78 (3.06)	—	—
12 weeks	17	8.82 (2.7)	16	8.81 (2.4)	0.02 (–1.34 to 1.38)	0.01
26 weeks	28	9.54 (2.91)	21	8.86 (3.29)	–0.69 (–2.12 to 0.75)	–0.23
Clinical measures						
UCLA^f^ Loneliness Scale						
0 weeks	46	47.96 (11.3)	26	48.04 (10.33)	—	—
12 weeks	30	45.23 (13)	22	44.14 (12.73)	–3.43 (–9.55 to 2.69)	–0.31
26 weeks	28	40.14 (13.23)	21	42.48 (12.38)	–1.44 (–7.64 to 4.77)	–0.13
PHQ-9^g^						
0 weeks	31	12.32 (5.06)	19	13.63 (5.11)	—	—
12 weeks	20	10 (6.1)	19	10.05 (4.89)	–1.72 (–5.46 to 2.02)	–0.34
26 weeks	21	8.9 (5.8)	18	9.83 (3.7)	0.54 (–2.92 to 4)	0.11
GAD-7^h^						
0 weeks	32	11.12 (4.77)	19	11.63 (4.93)	—	—
12 weeks	20	9.6 (6.2)	19	9.32 (4.77)	–2.36 (–5.58 to 0.85)	–0.50
26 weeks	21	8.1 (5.32)	18	8.28 (5.07)	1.08 (–2.64 to 4.81)	0.23

a SOFAS: Social and Occupational Functioning Assessment Scale.

b Not applicable.

c TUS: Time Use Survey.

d RMET: Reading the Mind in the Eyes Test.

e Weschler logical memory task.

f UCLA: University of California, Los Angeles.

g PHQ-9: Patient Health Questionnaire-9.

h GAD-7: Generalized Anxiety Disorder-7.

**Table 3.** Comparison of outcome data between participants who engaged for a minimum of 5 weeks and control participants.

	Intervention group (>5 weeks engagement)		Control group		Adjusted mean difference (95% CI)	Effect size (*d*)
	Participants, n	Mean (SD)	Participants, n	Mean (SD)		
Primary outcome measures						
Social and occupational function						
SOFAS^a^						
0 weeks	38	79.5 (8.3)	27	77.89 (8.86)	—^b^	—
12 weeks	27	81.22 (10.05)	22	78.45 (10.82)	–2.16 (–7.96 to 3.64)	–0.25
26 weeks	22	81.82 (10.15)	21	83.52 (7.7)	1.91 (–3.34 to 7.16)	0.22
TUS^c^ constructive economic activity						
0 weeks	38	46.27 (19.91)	27	45.3 (29.52)	—	—
12 weeks	28	35.6 (15.41)	22	48.32 (32.04)	11.76 (–1.12 to 24.64)	0.49
26 weeks	26	37.36 (20.04)	21	42.85 (14.91)	5.18 (–5.56 to 15.92)	0.21
TUS structured activity						
0 weeks	38	55.74 (19.82)	27	53.67 (28.23)	—	—
12 weeks	28	45.7 (16.36)	22	59.63 (31.38)	13.24 (0.73 to 25.75)	0.56
26 weeks	26	46.58 (21.41)	21	52.49 (16.09)	5.41 (–5.89 to 16.72)	0.23
Secondary outcome measures						
Cognitive and social cognitive function						
RMET^d^						
0 weeks	38	27.39 (4.48)	27	27.11 (3.48)	—	—
12 weeks	28	27.21 (4.48)	22	25 (4.16)	–0.87 (–2.66 to 0.93)	–0.21
26 weeks	26	28.08 (4.35)	21	27.48 (3.49)	0.13 (–1.79 to 2.04)	0.03
Logical memory^e^						
0 weeks	38	9.39 (3.12)	27	8.78 (3.06)	—	—
12 weeks	16	8.75 (2.77)	16	8.81 (2.4)	0.1 (–1.29 to 1.5)	0.03
26 weeks	26	9.58 (2.97)	21	8.86 (3.29)	–0.7 (–2.18 to 0.78)	–0.23
Clinical measures						
UCLA^f^ Loneliness Scale						
0 weeks	37	48.41 (11.72)	26	48.04 (10.33)	—	—
12 weeks	28	46.32 (12.67)	22	44.14 (12.73)	–4.4 (–10.51 to 1.72)	–0.40
26 weeks	26	40.54 (13.47)	21	42.48 (12.38)	–1.23 (–7.58 to 5.11)	–0.11
PHQ-9^g^						
0 weeks	25	12.88 (5.15)	19	13.63 (5.11)	—	—
12 weeks	19	10.32 (6.09)	19	10.05 (4.89)	–2.23 (–5.94 to 1.47)	–0.44
26 weeks	19	9.05 (6.07)	18	9.83 (3.7)	0.38 (–3.29 to 4.05)	0.07
GAD-7^h^						
0 weeks	26	11.58 (4.47)	19	11.63 (4.93)	—	—
12 weeks	19	9.53 (6.38)	19	9.32 (4.77)	–2.13 (–5.32 to 1.06)	–0.47
26 weeks	19	8.37 (5.47)	18	8.28 (5.07)	1.1 (–2.83 to 5.03)	0.24

a SOFAS: Social and Occupational Functioning Assessment Scale.

b Not applicable.

c TUS: Time Use Survey.

d RMET: Reading the Mind in the Eyes Test.

e Weschler logical memory task.

f UCLA: University of California, Los Angeles.

g PHQ-9: Patient Health Questionnaire-9.

h GAD-7: Generalized Anxiety Disorder-7.

#### Primary Outcome Measures: Social and Occupational Functioning

No difficulties were encountered in administering the SOFAS. In terms of group comparisons, there was a small effect on SOFAS scores at 12 weeks for those allocated to MOST (*d*=−0.28), with an equivalent effect size (*d*=−0.25) observed when only those who engaged for more than 5 of the 26 weeks (ie, >20%) were included in the intervention arm. Of note, there was no evidence of an effect of MOST on the SOFAS when measured at 26 weeks for either the full intervention group or for those who were at least minimally engaged.

Two issues emerged in the administration of the TUS. The first of these was COVID-19 related. Across the first 18 months of the recruitment period, time use was significantly altered by restrictions imposed due to the pandemic. The second was the issue of tracking activity over 6 months when students’ activity differed significantly depending on when they were in college during the semester, or during the holiday period between semesters. Consequently, the changes in TUS scores were problematic to interpret in terms of the size of the effect of the intervention.

#### Secondary Outcome Measures

##### Cognitive and Social Cognitive Assessment

As widely used cognitive measures, no difficulties were observed in administering either the logical memory task or the RMET. A small effect was observed on the RMET task at 12 weeks for both the intervention group (*d*=−0.23) and when only those who had at least minimally engaged were assessed (*d*=−0.21). No effect was observed on the declarative memory scale at 12 weeks. While a small effect at 26 weeks was observed in the full group (*d*=−0.23), this was not consistent between the full and the >20% engaged group.

##### Clinical Functioning

As already noted, the trial protocol was amended after 6 months to also include the GAD-7 and PHQ-9, which were therefore available for 51 of the 74 participants, 32 in the intervention arm and 19 in the control arm. Across the clinical measures, small to medium effects in favor of the intervention arm were observed at 12 weeks, with effect sizes of *d*=−0.5 for the GAD-7, *d*=−0.34 for the PHQ-9, and *d*=−0.31 for the Loneliness Scale. Comparable effect sizes were observed in the full intervention arm and when only those with >20% engagement were used in the comparison. Again, as with other effect sizes favoring the intervention arm, these effects were not observed at 26 weeks.

## Discussion

### Overview of Findings

This trial investigates the feasibility of conducting a randomized controlled trial of a moderated online intervention in a university setting. The intervention included online tailored mental health content with support from a therapist and peer-to-peer social mentoring and networking with the aim of improving mental health and social functioning. Based on recruitment at a single site, we achieved our recruitment target of 1.67 participants per month (~3.1 participants per semester month). Retention in the trial was 70% (52/74) at 12 weeks, reducing to 66% (49/74) at 26 weeks. For the intervention group, when engagement was measured in terms of participation in at least one component of the intervention (therapy journey, therapist contact, community wall participation, or peer support contact), 81% (38/47) of the intervention group engaged for 5 or more weeks of the trial (equivalent to at least 20% of the maximum 26 weeks for which the intervention was available to participants).

While the study was not adequately powered to test for differences between intervention and control groups, calculation of effect sizes associated with mean differences between the intervention group and the control arm indicated a small benefit favoring the intervention group (*d*=0.28) for the SOFAS measure of social and occupational functioning (but not the TUS). Similar small effects were observed for the secondary cognitive variables of memory function and social cognitive function. Finally, slightly larger (positive) effects were observed on the clinical measures available (*d*=−0.5 for GAD-7, *d*=−0.34 for PHQ-9, and *d*=−0.31 for the Loneliness Scale). Across each of these primary and secondary measures, the effect sizes observed at 12 weeks were similar when either the full intervention group or only those with at least minimal engagement (5 or more weeks) were compared to the control group. However, these effect sizes reduced to less than *d*=0.1 at 26 weeks.

### Progression to a Full Trial

As noted above, rates of recruitment were as originally planned, and no difficulties were encountered with randomization procedures and completion of outcome measures (except for the TUS, discussed further below). Retention rates in the trial of 70% (52/74) at 12 weeks were marginally lower than the criteria of 75%, indicating that fewer participants (n=4) were retained than expected. Our criteria of 75% was based on Alvarez-Jimenez et al’s [4] original study in patients with early psychosis. As such, this may have been unrealistic for a student population given that previous studies of MOST in a similar sample reported a retention rate of 59% [7].

### Outcome Measures Used

From the point of view of measurement of outcomes, our primary outcome was social and occupational functioning. Social and occupational function is difficult to measure accurately at the best of times [25]. Additionally, our study coincided with the COVID-19 pandemic, which significantly impacted social and occupational function and time use for many participants during the study. Furthermore, given the student experience of moving between the routine of term time and the social and occupational upheaval associated with the winter and summer breaks, intervention-related changes in functioning were difficult to track accurately. While observer-rated measurement using the SOFAS was able to detect the same level of effects as observed on cognitive and clinical measures, time use did not appear to be a sensitive or reliable measure of change in function. On this basis, other measures of social function might be considered to index change in this domain in a future trial. Qualitative feedback from participants would also be useful to give insight into how social function could best be captured in this sample.

Among the measures recorded in the study, the largest effect observed was on the GAD-7, a measure of generalized anxiety (*d*=−0.5). While this was not a primary outcome measure in the trial, measures of anxiety (and mood) are the measures most closely related to the therapy content of MOST given that much of the MOST therapy journeys focus on anxiety, social anxiety, or mood [13]. It might be expected, therefore, that the largest effects might be observed on these more proximal outcome measures.

A noticeable difference in the effect sizes can be observed across time points, with some benefits associated with participating in the intervention arm at 12 weeks and not appearing at 26 weeks. In the Alvarez-Jimenez et al [4] psychosis sample, no difference in social functioning was apparent between groups at follow-up, whereas a difference in the odds of enrolling in education or finding employment was observed. Given the near full college enrollment in our sample (as a study of students), we were obviously unlikely to see the same educational or occupational benefits. In the Van Doorn et al [7] study, the significant difference in social and occupational function, as measured by the SOFAS, did persist at 26 weeks. However, that study represented a single-group design investigating changes within the intervention group over time, as opposed to between-group differences. In this study, it is unlikely that these differences between 12 and 26 weeks are explained by engagement attrition, as most of the attrition occurred in the initial couple of weeks, with 83% (39/47) engaged for at least 5 weeks and 74% (35/47) engaged beyond 12 weeks. Instead, the reduced effect at 26 weeks appears to have reflected the improved scores of the control group at 26 weeks, as indexed by the SOFAS, the social cognitive, and clinical measures. Finally, implementing MOST after participants have attended counseling sessions represents a step-down approach designed to maintain positive treatment gains begun in the initial treatment received [23,44]. The findings at 12 weeks suggest that this maintenance of gains was achieved in this study. However, the length of the intervention and the level of engagement needed to continue improvement warrant further investigation. This is discussed below.

### Strengths, Limitations, and Future Directions

The purpose of a pilot study is to identify potential difficulties and avoid these prior to commencing a full trial. In terms of strengths, several aspects of the methodology for evaluating the use of MOST in a young adult student sample were supported in this study, including the feasibility of recruitment and retention, randomization, and a majority of the assessments used at each time point. In terms of the weaknesses of our methodology, we have already noted the difficulty with measuring social and occupational function. Specifically, the TUS might not be a suitable measure for use in a student population. We have also noted that the largest effect sizes for MOST are likely to be observed on clinical outcome measures that relate more directly to the intervention. As previously outlined, the addition of some of the clinical measures came after the trial had begun, and thus, data were missing for some participants. While the rationale for the inclusion of these variables is sound (see outcomes used in other MOST trials as well as digital interventions in this context [3,7,18]), and the results are promising, further evidence is needed to examine the impact of MOST on these outcomes.

We also note that the dropout rate was slightly higher in the intervention arm compared to the control arm. This is likely to reflect, in part, the time demands of participating in the various components of MOST. One unanswered question following this study is how long students should be expected to participate. As noted in Multimedia Appendix 3, there were clear significant differences between participants in terms of their interest or willingness to remain involved across the 26 weeks that MOST was offered. While, as noted, the vast majority remained active for more than 5 weeks, the median number of months of involvement was 5 months (mean 4.32, SD 1.95). This information should inform expectations for involvement in the full trial.

In terms of the implementation of the intervention, fidelity checklists were not used in this trial. A future evaluation of MOST in this context would benefit from adopting a fidelity procedure to ensure consistency in the delivery of the intervention. Such a checklist is being adopted by Mangelsdorf et al [45] in their recently begun trial of MOST for young people with depressive symptoms.

In line with best practice in randomized trials, this study examined the feasibility of comparing participants using MOST with care-as-usual participants. While this approach in a future full trial will be vital for investigating the effectiveness of MOST, it would also be useful to compare MOST to other existing digital mental health interventions. Given the rise in interest in digital interventions both generally and in the university context (see [3,18]), such a comparison would give insight both to the comparative effectiveness of MOST and would allow for further exploration of attrition rates in MOST, as well as barriers and facilitators to engagement.

As reported above, 72% (53/74) of the sample identified as female. Literature around the prevalence of mental health difficulties and around help-seeking in young people indicates that more females present with and seek help for mental health difficulties at university than males [21,46]. However, this overrepresentation of females in the sample limits the generalizability of the results and should be addressed in future studies of MOST.

Other limitations in this study include potential self-selection bias in terms of participation in the intervention and completion of assessments. As noted in the participant flowchart (Figure 1), 13 young people declined to participate in MOST, and attrition in the assessments was higher than in the intervention itself, with 39 participants continuing to engage with MOST at 12 weeks, but only 30 opting to complete assessments at this time. It was also outside the scope of this study to report on findings beyond 26 weeks. Further follow-up with participants would not only inform a future trial but would also give insight in terms of the long-term efficacy of MOST. Finally, this trial was impacted by the COVID-19 pandemic. As mentioned, this had an impact on the social and occupational functioning of participants. Furthermore, some assessments took place online due to restrictions, and the pandemic may have had an impact on engagement with MOST, with participants potentially engaging differently than they might have otherwise. Further examination of engagement with MOST is thus warranted.

### Conclusions

Based on the recruitment, retention, and engagement rates observed, this pilot feasibility study provides evidence for the feasibility of a full randomized controlled trial of MOST with a young adult population. Moreover, the effect sizes favoring the intervention arm are consistent with previous studies, suggesting that MOST may be a potentially beneficial support for youth mental health in the context of further education. This study also highlights important factors that need to be addressed in a full study, such as including measures of anxiety and depression as potentially primary outcome variables, of selecting sensitive measures of social function, and of ensuring sustained engagement in the intervention.

## Supplementary material

10.2196/73269 Multimedia Appendix 1 Recruitment figures per month across the study period.

10.2196/73269 Multimedia Appendix 2 Summary of engagement on MOST (Moderated Online Social Therapy).

10.2196/73269 Multimedia Appendix 3 Engagement on MOST (Moderated Online Social Therapy) by month.

10.2196/73269 Checklist 1 CONSORT checklist.

## Abbreviations

ANCOVA: analysis of covariance
AUDIT: Alcohol Use Disorders Identification Test
CBT: cognitive behavioral therapy
CONSORT: Consolidated Standards of Reporting Trials
DSM-IV: *Diagnostic and Statistical Manual of Mental Disorders, Fourth Edition*
DUDIT: Drug Use Disorders Identification Test
EIP: Early Intervention for Psychosis
GAD-7: Generalized Anxiety Disorder-7
GDPR: General Data Protection Regulation
MOST: Moderated Online Social Therapy
PHQ-9: Patient Health Questionnaire-9
PSYcHE: Improving Psychosocial Supports in Youth Mental Health
RMET: Reading the Mind in the Eyes Test
SOFAS: Social and Occupational Functioning Assessment Scale
TUS: Time Use Survey
UCLA: University of California, Los Angeles
