# Supplementary figures and images for: Moderated Online Social Therapy (MOST) in Help-Seeking Young People: Pilot Randomized Controlled Study
Source: J Med Internet Res. 2025 Nov 21;27:e73269. doi: 10.2196/73269 (PMC12638037; doi:10.2196/73269)

# Recruitment figures per month across the study period


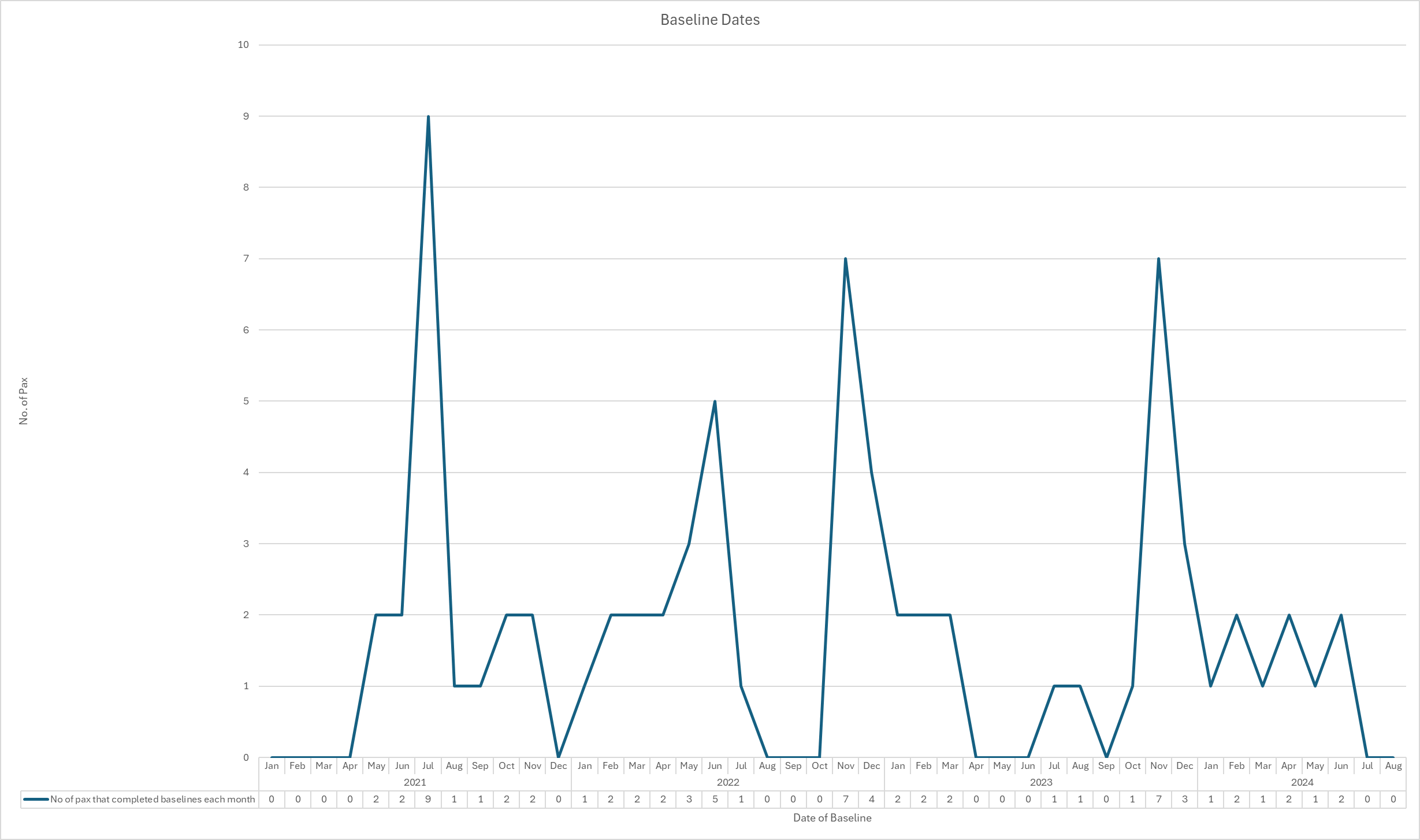

Supplement: Multimedia Appendix 1 [file jmir-v27-e73269-s001.docx]
