# Supplementary material for: Moderated Online Social Therapy (MOST) in Help-Seeking Young People: Pilot Randomized Controlled Study
Source: J Med Internet Res. 2025 Nov 21;27:e73269. doi: 10.2196/73269 (PMC12638037; doi:10.2196/73269)
Supplement: Multimedia Appendix 2 [file jmir-v27-e73269-s002.docx]

# Summary of engagement on MOST (Moderated Online Social Therapy)

|  | *Min* | *Max* | *Median* | *M (SD)* |
| --- | --- | --- | --- | --- |
| Total activity time (mins) | 0 | 249 | 46 | 70.94 (69.81) |
| Journey activities completed | 0 | 96 | 16 | 24.94 (22.48) |
| Explore activities completed | 0 | 20 | 0 | 1.45 (3.53) |
| Total activities completed | 0 | 98 | 17 | 24.94 (24.02) |
| Posts made | 0 | 17 | 1 | 1.62 (3.1) |
| Posts commented on | 0 | 30 | 0 | 1.87 (4.62) |
| Posts reacted to | 0 | 36 | 0 | 3.28 (7.74) |
| PSW calls | 0 | 10 | 2 | 2.91 (2.77) |
| Therapist calls | 0 | 11 | 1 | 2.66 (2.71) |
| Engagement (months) | 0 | 6 | 5 | 4.32 (1.95) |
