# Supplementary material for: Moderated Online Social Therapy (MOST) in Help-Seeking Young People: Pilot Randomized Controlled Study
Source: J Med Internet Res. 2025 Nov 21;27:e73269. doi: 10.2196/73269 (PMC12638037; doi:10.2196/73269)
Supplement: Multimedia Appendix 3 [file jmir-v27-e73269-s003.docx]

# Engagement on MOST (Moderated Online Social Therapy) by month

|  |  | Total  (*N* = 47) |
| --- | --- | --- |
| Total number of participants who engaged in… | | |
|  | Month 1 | 42 |
|  | Month 2 | 39 |
|  | Month 3 | 36 |
|  | Month 4 | 29 |
|  | Month 5 | 29 |
|  | Month 6 | 28 |
| Total number of participants who remained engaged in said month and those prior | | |
|  | Month 1 | - |
|  | Month 2 | 37 |
|  | Month 3 | 33 |
|  | Month 4 | 28 |
|  | Month 5 | 22 |
|  | Month 6 | 20 |
| Total number of participants who disengaged in said month but re-engaged later | | |
|  | Month 1 | 2 |
|  | Month 2 | 2 |
|  | Month 3 | 3 |
|  | Month 4 | 6 |
|  | Month 5 | 3 |
|  | Month 6 | - |
| Total number of participants who completely disengaged in… | | |
|  | Month 1 | 3 |
|  | Month 2 | 2 |
|  | Month 3 | 2 |
|  | Month 4 | 3 |
|  | Month 5 | 3 |
|  | Month 6 | 2 |
